# Insomnia Symptoms in Doctor of Pharmacy Students in the United States: A Multicenter Study

**DOI:** 10.7759/cureus.116006

**Published:** 2026-09-09

**Authors:** Lamisa Hossain, Katie Nguyen, Kristine Le, Farah Khorassani

**Affiliations:** 1 Clinical Pharmacy Practice, University of California Irvine School of Pharmacy and Pharmaceutical Sciences, Irvine, USA

**Keywords:** higher education, insomnia, mental health, pharmacy students, student wellness

## Abstract

Introduction: Insomnia is a common sleep disorder that can negatively affect academic performance, mental health, and quality of life. Despite the rigor of Doctor of Pharmacy (PharmD) training, little is known about the prevalence of insomnia among PharmD students in the United States. This study evaluated the presence of insomnia across multiple institutions and characterized symptoms to inform targeted interventions. Materials and methods: This is a cross-sectional, institutional review board-exempt survey of pharmacy students at nine schools in the United States between March and April 2025. Participants completed demographic information and the Insomnia Symptom Questionnaire (ISQ), a validated 13-item instrument designed to detect insomnia. The primary outcome was the proportion of students meeting ISQ criteria for symptoms consistent with insomnia. Secondary outcomes included characterization of insomnia-related symptoms and comparisons by year in training. Descriptive statistics and chi-squared tests were applied. Results: Seventy-three students completed the survey (mean age 25.5 years; 59% female; 74% Asian American). Overall, 26% met criteria for symptoms consistent with insomnia, with the highest prevalence among first-year (35%) and fourth-year (27.3%) students. Fatigue was the most common symptom, reported by 53.4% of students, followed by daytime sleepiness (38.3%) and concentration difficulties (37%). Difficulty staying asleep and frequent awakenings were less often endorsed. Conclusions: Approximately one in four PharmD students screened positive for symptoms consistent with insomnia, with fatigue and difficulty concentrating as prominent features. These findings highlight a potential need for wellness initiatives and targeted interventions to support sleep health in pharmacy education.

## Introduction

This article was previously presented as a meeting abstract at the 2026 American Association of Psychiatric Pharmacy Conference on April 20, 2026. Insomnia is characterized by self-reported difficulty with sleep [1]. In the American Psychiatric Association's Diagnostic and Statistical Manual of Mental Disorders, the primary diagnostic criterion for insomnia includes the complaint of unsatisfying sleep quantity/quality associated with at least one of the following symptoms: difficulty initiating sleep, maintaining sleep, or returning to sleep [2,3]. According to an undergraduate study performed by Buboltz and colleagues, insomnia is a common sleep disorder that can have consequences for daytime functioning in college-aged students [4]. These include CNS symptoms such as increased tension, irritability, depression, and confusion. Insomnia can also reduce quality of life and, among students, may contribute to poorer academic performance due to lack of sleep. Lack of sleep may also impair cognitive and physical functioning and is associated with a wide range of impairments in daytime functioning across several emotional, social, and physical domains [5]. Further, poor sleep has been associated with impaired studying and classroom performance [6,7].

To date, the prevalence of insomnia in pharmacy students has not been well described. One cross-sectional study conducted among Saudi Arabian pharmacy students found that 43% of respondents met the criteria for insomnia when assessed using a non-validated sleep questionnaire [7]. Although this study evaluated pharmacy students, it was conducted at a single center outside the United States.

The primary aim of this study was to assess the prevalence of insomnia symptoms among PharmD students across institutions in the United States using the Insomnia Symptom Questionnaire (ISQ), a validated insomnia rating scale. Secondarily, we aimed to characterize sleep complaints and describe differences in the prevalence of insomnia based on year of study. We hypothesized that insomnia symptoms would be prevalent in PharmD students. Our goal is to produce actionable data to justify targeted interventions for PharmD students to combat the deleterious effects of disrupted sleep.

## Materials and methods

Study design

This was an institutional review board-exempt, cross-sectional survey study using convenience sampling and voluntary participation, including all students willing to respond from pharmacy schools in California and New York. Individuals who were not enrolled in a pharmacy program were excluded. The survey was disseminated through multiple channels, including email distribution, a QR code distributed at Western Pharmacy Exchange and in classrooms, an announcement in the institutional newsletter, social media, and direct verbal outreach. The survey link was available for approximately four weeks, from March 31, 2025, to April 25, 2025. Response rates were not calculated due to the survey's open dissemination.

The optional survey was distributed using Google Forms (Google LLC, Mountain View, CA, USA). All participants remained anonymous and were assigned a participant ID. Demographic questions, such as age, gender, and ethnicity, along with weekly work hours (internship or other employment) and comorbid psychiatric conditions, were collected to characterize study participants. The ISQ is a 13-item self-administered questionnaire used to identify symptoms of insomnia in participants; it assesses the severity and frequency of sleep difficulties, sleep complaints, and daytime symptoms associated with sleep impairment [8]. The authors of this paper received permission from the study authors to use this instrument for research. The ISQ questions were derived from the American Psychiatric Association’s DSM-IV-TR criteria for primary insomnia and are aligned with the American Academy of Sleep Medicine's Research Diagnostic Criteria [8]. Full details on the development, scoring, and psychometric properties are available in the original validation report [8]. Although the ISQ was originally validated in middle-aged women with insomnia, it was used for the present study because women comprise the majority of PharmD students and the instrument assesses general insomnia symptoms. Insomnia symptoms were assessed using the ISQ, which was scored according to the published scoring algorithm [8]. Participants meeting the established ISQ criteria were classified as having symptoms consistent with insomnia.

Outcomes

The primary outcome was the percentage of PharmD students who met the ISQ criteria for symptoms consistent with insomnia. This was determined using the ISQ scoring scale. To screen positive for symptoms consistent with insomnia, participants were required to report "frequently" or "always" for at least one of the following: difficulty falling asleep, difficulty staying asleep, or unrefreshing sleep. They were also required to report a duration of ≥4 weeks for at least one sleep difficulty and to rate at least one daytime impairment item (questions 6-13) as "quite a bit" or "extremely." The secondary outcomes were to characterize the individual symptoms reported and to compare insomnia prevalence across years in the PharmD program.

Statistical analysis

Descriptive statistics were performed to characterize participant demographics. Categorical variables were compared using Pearson's chi-square test or Fisher's exact test, as appropriate for sample size. Statistical significance was defined as p < 0.05. No institutional clustering was performed.

## Results

Seventy-three PharmD students from nine PharmD schools were included in the analysis. Students from the University of California, Irvine School of Pharmacy and Pharmaceutical Sciences comprised 71% (n = 52) of the study population. Most participating students were female (n = 43, 58.9%) (Table 1). The largest ethnic group represented was Asian American participants (n = 54, 74%), and the mean age was 25.5 years. Seventy-eight percent (n = 57) of students reported no mental health diagnosis at the time of the survey. However, co-occurring major depressive disorder and generalized anxiety disorder were the most frequently reported psychiatric conditions. Seventy-one percent (n = 52) of students reported working shifts of 8-32 hours per week.

**Table 1:** Participant demographics

	Total, n (%)
Age (years), mean	25.5
Gender, n (%)	
Female	43 (58.9)
Male	29 (39.7)
Prefer not to say	1 (1.4)
Ethnicity, n (%)	
Asian American/Asian	54 (74)
Caucasian/White	6 (8.2)
Middle Eastern or North African	4 (5.5)
Hispanic/Latinx	3 (4.1)
Multi-race	5 (6.8)
Missing	1 (1.4)
Substance use, n (%)	
None	38 (52.1)
Yes, alcohol	25 (34.2)
Yes, recreational drugs	0 (0)
Yes, alcohol and recreational drugs	10 (13.7)
Pre-existing conditions, n (%)	
None	57 (78.1)
Major depressive disorder	1 (1.4)
Generalized anxiety	6 (8.2)
Both	6 (8.2)
Prefer not to say	3 (4.1)
Hours worked per week, n (%)	
0 h	12 (16.4)
1-8 h	18 (24.7)
8-16 h	26 (35.6)
16-32 h	15 (20.5)
32-40 h	1 (1.4)
>40 h	1 (1.4)

Results of the primary endpoint analysis indicate that 26% (n = 19) of students screened positive for insomnia (Table 2).

**Table 2:** ISQ-defined insomnia by PharmD year PharmD: Doctor of Pharmacy, PY: pharmacy year, ISQ: Insomnia Symptom Questionnaire

Pharmacy year	ISQ-defined insomnia, n (%)
PY1 (n = 20)	7 (35)
PY2 (n = 31)	7 (22.6)
PY3 (n = 11)	2 (18.2)
PY4 (n = 11)	3 (27.3)
Total (n = 73)	19 (26)

Rates were highest among first-year (35%, n = 7) and fourth-year (27.3%, n = 3) PharmD students. Given the small number of respondents in some classes, Fisher’s exact test was used, and the results indicated no statistically significant difference in insomnia rates between classes (p = 0.740). Eighty percent (n = 15) of participants who satisfied the ISQ criteria for symptoms consistent with insomnia reported using alcohol and/or recreational drugs.

When evaluating responses to individual survey questions, the most frequently reported sleep symptom was fatigue, with 53.4% (n = 39) reporting that it was quite a bit or always present (Table 3).

**Table 3:** ISQ responses by question ISQ: Insomnia Symptom Questionnaire

Criterion	Interview question (during the past month)	None (not present at all)	Rarely (present a little bit)	Sometimes (present moderately)	Frequently (present quite a bit)	Always (present always or extremely)
		n (%)	n (%)	n (%)	n (%)	n (%)
Sleep symptom/duration	(1) Difficulty falling asleep	23 (31.5)	22 (30.1)	17 (23.3)	9 (12.3)	2 (2.8)
Sleep symptom/duration	(2) Difficulty staying asleep	46 (63)	13 (17.8)	8 (11)	4 (5.5)	2 (2.7)
Sleep symptom/duration	(3) Frequent awakenings from sleep	34 (46.6)	24 (32.9)	7 (9.6)	5 (6.8)	3 (4.1)
Sleep symptom/duration	(4) Feeling that your sleep is not sound	30 (41.1)	19 (26)	16 (21.9)	7 (9.6)	1 (1.4)
Sleep symptom/duration	(5) Feeling that your sleep is unrefreshing	19 (26)	18 (24.7)	24 (32.9)	7 (9.6)	5 (6.8)
Daytime impairment	(6) How much do your sleep problems bother you?	16 (21.9)	23 (31.5)	21 (28.8)	11 (15.1)	2 (2.7)
Daytime impairment	(7) Have your sleep difficulties affected your work?	16 (21.9)	26 (35.6)	19 (26)	8 (11)	4 (5.5)
Daytime impairment	(8) Have your sleep difficulties affected your social life?	21 (28.8)	21 (28.8)	21 (28.7)	8 (11)	2 (2.7)
Daytime impairment	(9) Have your sleep difficulties affected other important parts of your life?	20 (27.4)	27 (37)	16 (21.9)	7 (9.6)	3 (4.1)
Daytime impairment	(10) Have your sleep difficulties made you feel irritable?	18 (24.7)	15 (20.5)	15 (20.6)	20 (27.4)	5 (6.8)
Daytime impairment	(11) Have your sleep problems caused you to have trouble concentrating?	13 (17.8)	13 (17.8)	20 (27.4)	17 (23.3)	10 (13.7)
Daytime impairment	(12) Have your sleep difficulties made you feel fatigued?	10 (13.7)	10 (13.7)	14 (19.2)	26 (35.6)	13 (17.8)
Daytime impairment	(13) How sleepy do you feel during the day?	2 (2.8)	13 (17.8)	30 (41.1)	22 (30.1)	6 (8.2)

Daytime sleepiness was reported as occurring frequently or always by 38.3% (n = 28) of students. Thirty-seven percent (n = 27) reported that sleep problems caused difficulty concentrating "frequently" or "always." Difficulty staying asleep was not reported or was rarely reported by most students (n = 59, 80.8%). Frequent awakenings were not reported or were rarely reported in 79% (n = 58) of participants.

## Discussion

Among PharmD students at nine institutions in their first through fourth years, approximately one-quarter met criteria for symptoms consistent with insomnia when surveyed using the ISQ. This study was intended to screen for insomnia symptoms in PharmD students, not to diagnose, and should be interpreted with caution. Results from this analysis provide useful information on the prevalence of insomnia symptoms, as well as specific symptoms observed more frequently, such as daytime fatigue and difficulty concentrating, which could affect student learning and performance. Understanding symptom trends across different cohorts provides valuable data for planning and designing interventions to address sleep problems among students and improve overall well-being.

Of the participants who satisfied the ISQ criteria for symptoms consistent with insomnia, 80% reported using alcohol and/or recreational drugs. Although no formal sensitivity analysis was performed, the association between substance use and insomnia symptoms is an important area for future research since alcohol or recreational drugs are known to interfere with sleep architecture and could impact individuals’ ability to fall or stay asleep [3]. In a large sample of nearly 15,000 French university students, insomnia was more common in daily and weekly cannabis users, and daily cannabis use was associated with a twofold higher risk of experiencing insomnia [9]. Further inquiry into the degree of alcohol and recreational drug use is necessary to fully ascertain the impact on student sleep.

Out of the 19 participants who met criteria for ISQ-defined insomnia, 15 of them were Asian American participants (79%). Sleep disparities have been reported among Asian Americans, including shorter sleep duration and poorer sleep quality [10]. This raises important questions about insomnia across cultural groups, particularly among Asian American students, and whether familial, scholastic, or cultural constraints may contribute to factors influencing sleep, such as stress [10]. Despite these considerations, the disproportionate representation of Asian American participants in our sample limits our ability to determine whether their sleep is uniquely affected compared with that of a more diverse population.

In the United States, sleep among pharmacy students has been assessed using the Pittsburgh Sleep Quality Index [11]. In a survey of 253 pharmacy students in their first through third professional years, those in the didactic portion of their training experienced poor sleep quality [11]. Our results are generally consistent with those observations. The first-year cohort demonstrated the highest rate of ISQ-defined insomnia of all cohorts. Though institutional variation exists, the first year traditionally involves a heavy didactic course load. This survey also noted that lower GPA was associated with poorer sleep. Lack of sleep can affect clinical decision-making, memory consolidation, and learning for students [12]. Prolonged sleeplessness can be detrimental to academic performance, long-term professional competency, and mental health, especially for students in professional programs, such as PharmD students, who are expected to meet high standards and cognitive demands [12]. A study by Thabit and colleagues observed correlations between Saudi Arabian pharmacy students’ lack of sleep before exams and frequent daytime napping, both of which were associated with course failure in the prior year [7]. This further underscores the importance of improving sleep habits among PharmD students to optimize academic performance.

Poor PharmD student sleep can be addressed in several ways: more robust student wellness support, including access to behavioral health resources, educational programs on sleep, and culturally responsive counseling services, may be beneficial. Student support programs, such as the Sleep Treatment and Education Program for Students, have been described as helping students improve sleep [12]. The intervention included a 30-minute presentation with handouts covering sleep hygiene, stimulus control, and information on substances that impact sleep [13]. At the six-week follow-up, students who received the intervention demonstrated reduced sleep difficulties and improved sleep habits [13].

Other potential interventions could target efficient study habits and assistance with time management. Students may benefit from assistance with study habits to avoid last-minute studying and late nights that could affect sleep before academic assessments. An emphasis on time management skills may be especially useful for PharmD students. PharmD students are unique among doctoral students in that a substantial percentage hold internship positions and are expected to work long hours outside the classroom. In our sample, 70.8% of students worked between 8 and 32 hours per week (Table 1). These additional work responsibilities may interfere with healthy study and sleep habits; therefore, students may benefit from targeted interventions to improve sleep and academic performance.

Strengths of this study include the use of a validated screening instrument, the multi-institutional scope of the project, and secondary exploration into specific insomnia symptoms experienced by PharmD students in the United States. Limitations of this study include the small sample size and insufficient statistical power for adequate subgroup analyses, the cross-sectional design, limited narrative information to support survey responses, incomplete information regarding comorbid or undiagnosed psychiatric conditions other than major depressive disorder and generalized anxiety disorder, concurrent cannabis use, and limited ethnic diversity. Additionally, institutional clustering was not performed, and a large portion of the sample was from a single institution. Selection and nonresponse bias may also have occurred due to the survey's voluntary nature. Future studies are warranted to monitor responses longitudinally across different stages of pharmacy training and to identify specific factors that may contribute to poor sleep in more ethnically and geographically diverse student populations, thereby enabling targeted, time-specific mental health interventions.

## Conclusions

This multi-institutional, cross-sectional study highlights sleep-related concerns among PharmD students. Approximately one-quarter of PharmD students reported symptoms consistent with insomnia. The most prominent symptoms reported were daytime fatigue and difficulty concentrating. These symptoms may affect student well-being and academic performance, suggesting that further examination of these trends in a more ethnically and geographically diverse population is warranted. This also underscores an opportunity for guided, focused interventions to mitigate the effects of sleep loss among PharmD students with sleep complaints.
